# Highly efficient triazolone/metal ion/polydopamine/MCM-41 sustained release system with pH sensitivity for pesticide delivery

**DOI:** 10.1098/rsos.180658

**Published:** 2018-07-11

**Authors:** Huayao Chen, Guozhi Huang, Hongjun Zhou, Xinhua Zhou, Hua Xu

**Affiliations:** 1 School of Chemistry and Chemical Engineering, Zhongkai University of Agriculture and Engineering, Guangzhou, People's Republic of China; 2 Guangzhou Key Laboratory for Efficient Use of Agricultural Chemicals, Guangzhou, People's Republic of China

**Keywords:** polydopamine, MCM-41, sustained release, pH-sensitive, pesticide

## Abstract

MCM-41 was prepared through the sol–gel method and encapsulated by polydopamine (PDA) before being coordinated with metal ions to form a highly efficient sustained release system (M-PDA–MCM-41) for triazolone delivery. The characterization results confirmed the existence of the coordination bond between the PDA layer and triazolone through the bridge effect from metal ions, which enhanced the interaction between PDA–MCM-41 and triazolone. The adsorption capacity of Fe-PDA–MCM-41 increased up to 173 mg g^–1^, which was 160% more than that of MCM-41. The sustained release performance of M-PDA–MCM-41 in different pH values was investigated. Under the conditions of pH ≤7, the release speed of triazolone increased with pH decreasing, whereas its release speed in the weak base condition was slower than in the neutral condition. Therefore, the as-synthesized system showed significant pH-sensitivity in the sustained release process, indicating that the sustained release system can be well stored in the neutral or basic environment and activated in the acid environment. Their sustained release curves described by the Korsmeyer–Peppas equation at pH 7 showed the same behaviour, indicating that PDA decoration or metal ion coordination only increases the steric hindrance and the interaction between carrier and triazolone instead of changing the original structure of the pure MCM material in accordance with X-ray diffraction and Brunauer-Emmett-Teller analysis results.

## 1. Introduction

In the past decade, mesoporous silica materials have attracted the attention of pharmaceutical researchers around the world. As we know, mesoporous silica materials have many unique properties, such as their non-toxic nature, large surface area and pore volume, tuneable pore size, as well as chemically inert and easily modified surface properties [1–5]. After over a decade of booming development, mesoporous silica nanoparticles have been regarded as one of the most promising biomedical platforms for therapeutic, diagnostic, prognostic and combinatorial applications [6–8]. Benefiting from their stable mesoporous structures, large surface area, tuneable pore size, easy surface functionalization and good biocompatibility, mesoporous silica nanoparticles can not only be fine-tuned to achieve the desired physico-chemical characteristics for accommodating multiple cargo molecules such as therapeutic drugs, proteins, genes and imaging agents either alone or in combination, but also be engineered to facilitate the on-demand drug release and multimodality imaging [9–11]. Meanwhile, various types of such materials have been developed into drug delivery systems [12,13].

Drugs enter mesoporous silica mainly through adsorption, so the latter must be surface-modified to prevent drug leakage and to control drug release [14]. Polydopamine (PDA) is a biomimetic polymer which can form on a wide range of materials, including ceramics, copolymers and semiconductors, through oxidative polymerization in a weak alkaline condition (pH 8.0–8.5) [15–17]. PDA coating, as an excellent gatekeeper on the surface of mesoporous silica, is extremely sensitive to pH value. With PDA coating, drug molecules are easily blocked in mesoporous silica nanoparticles under neutral conditions and released at lower pH values [18,19]. However, the study in the sustained released system based on MCM-41 coated with PDA with pH-sensitivity for the pesticide delivery is rarely reported in spite of it being useful for pest control in agriculture [20–22].

Based on the research mentioned above, we proposed the pH-sensitive highly efficient PDA decorated triazolone/MCM-41 sustained release system for pesticide delivery, and the relationship between the PDA encapsulation with its adsorption and sustained released performance was also investigated. The sustained release performance and adsorption properties were further improved by coordination with metal ions which strengthened the interaction between the PDA layer and triazolone through the bridge effect. Finally, the Tri/M-PDA–MCM-41 sustained release system assembled by MCM-41 mesoporous silica, PDA layer, metal ions and triazolone with pH-sensitivity and larger adsorption capacity (AC) for pesticide delivery was developed which would thus be expected to bring positive effects in agricultural fields for pest control [23], especially for the acid soils in South China [24–26].

## 2. Material and methods

### 2.1. Chemicals

Cetyl trimethyl ammonium bromide (CTAB), tetraethyl orthosilicate (TEOS), ethanol, ammonia, ferric nitrate, zinc nitrate, copper nitrate, sodium hydroxide and hydrochloride were obtained from Tianjin Damao Chemical Reagents. Dopamine hydrochloride and tris(hydroxymethyl)methylaminomethane (THAM) were obtained from Jinchun Biochemical Co., Ltd. Triazolone (greater than 99%, Jiangsu Jinghong Chemical Engineering Co., Ltd.) was also used in this work. All chemicals were of analytical grade and used as received without any further purification.

### 2.2. Preparation of MCM-41

According to previous research [27], the sol–gel method was adopted to prepare MCM-41. A total of 2.0 g of CTAB, 100 ml of deionized water and 60 ml of ammonia were added to the flask to be dissolved at 60°C with stirring for 1 h. About 5 g of TEOS was added to the solution dropwisely for 6 h before being crystalized at room temperature for 3 days. Then, the sample was obtained after filtered, washed and dried. Finally, the template was removed by ethanol to attain MCM-41.

### 2.3. Preparation of polydopamine–MCM-41

Approximately 0.5 g of MCM-41, 100 mg of dopamine hydrochloride and 800 ml of THAM–HCl buffer solution (pH 8.5) were added to the flask to be dissolved at 25°C under stirring for 24 h. Then, PDA–MCM-41 was attained after being filtered, washed and dried.

### 2.4. Preparation of M-PDA–MCM-41

About 250 ml of copper nitrate, zinc nitrate and ferric nitrate solution (0.1 mol l^–1^) were added to 0.5 g of PDA–MCM-41 at 30°C under stirring for 24 h. Then, Cu-PDA–MCM-41, Zn-PDA–MCM-41 and Fe-PDA–MCM-41 were attained after being filtered, washed and dried in oven at 90°C for 24 h.

### 2.5. The loading of triazolone

The supported triazolone was prepared via impregnation. The mesoporous silicas were activated under vacuum at 80°C for 6 h, and 0.5 g of sample was immersed in 35 ml of triazolone ethanol solution (10 mg ml^–1^) at 35°C under stirring for 24 h, then filtered, washed and dried. The samples obtained were denoted as Tri/MCM-41, Tri/PDA–MCM-41 and Tri/M-PDA–MCM-41, respectively, according to the different carrier. The filter liquor was characterized by ultraviolet–visible spectrophotometer (UV–vis) to calculate the concentration after adsorption.

### 2.6. Adsorption properties test

A UV-2550 UV–vis spectrophotometer from Shimadzu Co., Japan was applied to measure the amount of triazolone adsorbed by mesoporous silica. Linear regression of the solution concentration (*C*/(mg × l^–1^)) and absorbance (*A*) of triazolone standard ethanol solutions of different concentrations at *λ* = 223 nm were performed to obtain a standard curvilinear equation: *C* = 40.00*A* + 9.220, *R*^2^ = 0.9993. UV spectroscopy was performed to measure the absorbance of this ethanol solution before and after the adsorption in triazonlone ethanol solution. AC and loading content (LC) may be calculated by the following equations:

2.1 $$\text{AC}=\frac{(C_{0}-C_{1})\times V}{m}$$

and

2.2 $$\text{LC}=\frac{(C_{0}-C_{1})\times V}{m\times \text{1000}+(C_{0}-C_{1})\times V},$$

where *C*_0_ is the origin mass concentration (mg l^–1^) of the triazolone in ethanol solution, *C*_1_ is the mass concentration (mg l^–1^) of the triazolone in ethanol solution after adsorption, and *m* is the mass (g) of mesoporous silica.

### 2.7. Sustained release performance test

The performance of sustained release triazolone particles was tested according to Chen *et al.* [28]. The (*M*_1_, mg) drug-loaded particles were weighed and placed in a conical flask filled with 50 ml of 50% ethanol. Linear regression of the solution concentration (*C*/(mg × l^–1^)) and absorbance (*A*) of triazolone standard solutions of different concentrations at *λ* = 223 nm were performed to obtain a standard curvilinear equation: *C* = 28.82*A* + 0.02248, *R*^2^ = 0.9993. At intervals of (*t*), 1 ml of the sample solution was transferred and diluted to 25 ml. An equal volume of the original sustained release solution was then added to the conical flask to replace the withdrawn sample. The absorbance of the 25 ml solution was obtained, and the cumulative release amount of triazolone was calculated as *R*_i_. A *R*_i_−*t* curve was drawn to study the release kinetics of triazolone. *R*_i_ may be calculated by the following equation:

2.3 $$R_{i}=\left\{ \begin{array}{l} \frac{\rho_{i}\times 0.1}{M_{1}\times \text{LC}}(i=1)\\ \frac{\rho_{i}\times 0.1}{M_{1}\times \text{LC}}+\frac{\sum_{i=1}^{i-1}\rho_{i}\times 0.002}{M_{1}\times \text{LC}}(i=2,3,4\ldots ), \end{array}\right.$$

where *ρ*_i_ is the mass concentration (mg l^–1^) of triazolone for each sampling.

### 2.8. Structural characterization of particles

The samples were analysed using a Bruker AXS D8 X-ray diffractometer (Bruker AXS GmbH, Karlsruhe, Germany) with Cu radiation (*λ* = 1.5418 Å) and a graphite monochromator at 25°C, 40 kV and 30 mA. The measurements were scanned at 2° min^−1^ (angular range 2*θ* = 0.5 – 10°) in 0.02° step size. X-ray photoelectron spectra (XPS) were recorded on a ESCALAB 250XI spectrometer (Thermo Fisher Scientific, Al K*α*, *hν* = 1486.6 eV) under a vacuum of ∼2 × 10^–7 ^Pa. Charging effects were corrected by adjusting the main C 1s peak to a position of 284.8 eV. The structure of the particles was analysed by a Spectrum100 Fourier infrared spectrometer (PerkinElmer Inc., USA) by using the potassium bromide squash technique. The gold particles were sprayed on the surface of samples under protection of N_2_ and the scanning electron microscopy (SEM) was observed by an S4800 scanning electron microscope (Hitachi, Japan) to observe the surface topography. Transmission electron microscopy (TEM) observation was conducted on a FEI Tecnai G2 F20 transmission electron microscope (FEI, USA). The Brunauer-Emmett-Teller (BET) surface area of samples was determined by N_2_ adsorption isotherms at 77 K, operated on Quadrasorb SI adsorption equipment (Quantachrome, USA). The samples were degassed at 200°C for 12 h in vacuum before the N_2_ adsorption experiment. The loading amount of metal ions was confirmed by inductively coupled plasma-atomic emission spectrometry (Agilent 725, USA).

## 3. Results and discussion

### 3.1. Structure characterization of mesoporous materials

**Figure 1.**
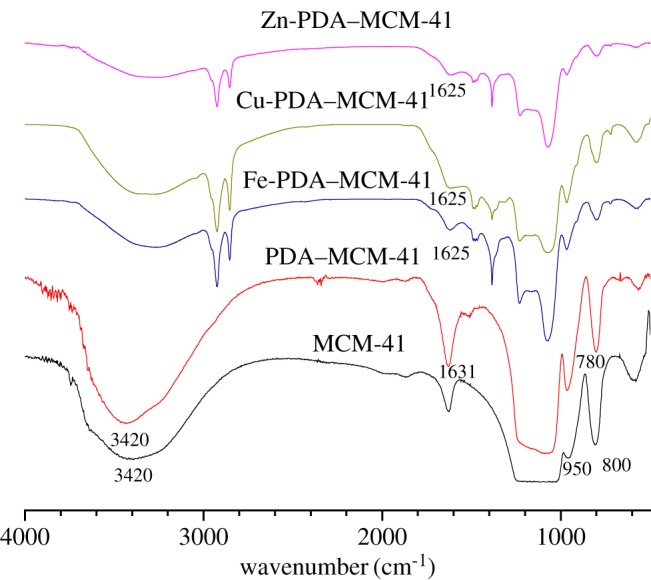
FTIR spectra of MCM-41, PDA–MCM-41, Zn-PDA–MCM-41, Cu-PDA–MCM-41 and Fe-PDA–MCM-41.

Fourier transform infrared spectroscopy (FTIR) was carried out to compare the different compositions of MCM-41, PDA–MCM-41, Zn-PDA–MCM-41, Cu-PDA–MCM-41 and Fe-PDA–MCM-41. As shown in figure 1, two bands appeared in 3420 cm^–1^ and 950 cm^–1^ for MCM-41 ascribed to the stretching and bending vibration of Si-OH, respectively [29,30]. The band at 800 cm^–1^ [31] was attributed to the characteristic peaks of Si–O–Si on the SiO_2_ framework. For the spectra of PDA–MCM-41, a new band at 780 cm^–1^ appeared belonged to the characteristic peak of ortho-double substituted aromatic rings, and the band of 3420 cm^–1^ ascribed to the hydroxy group of PDA was significantly enhanced which confirmed that the sample was encapsulated by PDA. The blue shift of the stretching vibration band from C=O from 1631 to 1625 cm^–1^ happened after the coordination with metal ions.

**Figure 2.**
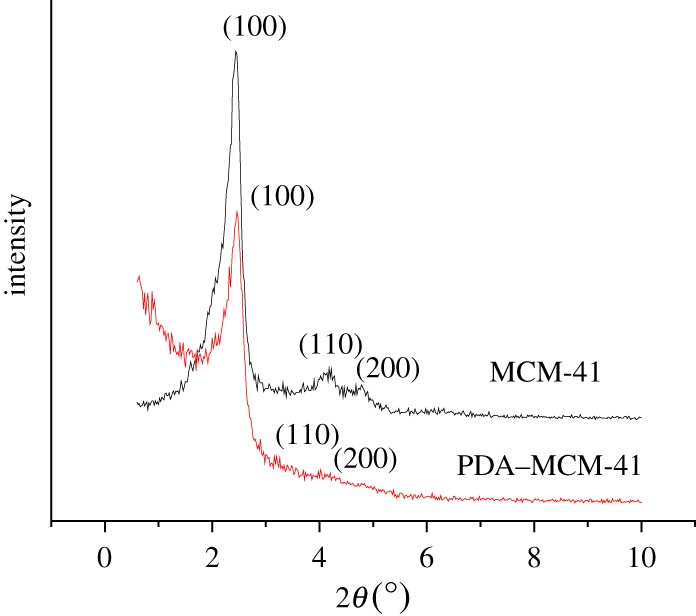
XRD patterns of MCM-41 and PDA–MCM-41.

Figure 2 shows the X-ray diffraction (XRD) patterns of MCM-41 and PDA–MCM-41. Three characteristic peaks of MCM-41 ascribed to the (100), (110) and (200) crystal face representing the regular hexagonal pore structure [32] still existed in the XRD patterns of PDA–MCM-41, indicating that the regular hexagonal pore structure remained after PDA encapsulation. The strength of the XRD peaks of the (100), (110) and (200) crystal face decreased after encapsulated by PDA, which convinced us that PDA was introduced to the system and decreased its degree of orderliness [33].

**Figure 3.**
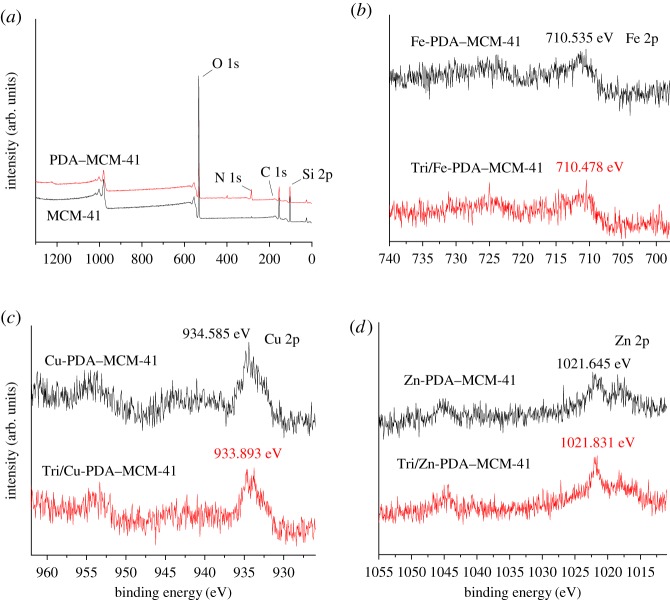
XPS spectra of MCM-41 and PDA–MCM-41 (*a*), Fe-PDA–MCM-41 and Tri/Fe-PDA–MCM-41 (*b*), Cu-PDA–MCM-41 and Tri/Cu-PDA–MCM-41 (*c*), Zn-PDA–MCM-41 and Tri/Zn-PDA–MCM-41 (*d*).

The XPS analysis was carried out to identify the surface elements chemical states, as shown in figure 3. After being encapsulated by PDA, the new binding energy (BE) of nitrogen appeared as shown in figure 3*a*, indicating that the mesoporous silica had been encapsulated by PDA. For Fe-PDA–MCM-41 and Zn-PDA–MCM-41 as shown in figure 3*b,d*, the BE of ferric and zinc decreased from 710.535 eV to 710.478 eV and increased from 1021.645 eV to 1021.831 eV, respectively, after loading triazolone owing to the electron transfer between the metal ions and triazolone which implied the coordination interaction formed between tiazolone and metal ions, which strengthened the interaction of the mesoporous silica carrier with triazolone. For Cu-PDA–MCM-41 as shown in figure 3*c*, the BE of copper has a significant shift from 934.585 to 933.893 eV after loading triazolone, indicating that the interaction between copper ion and triazolone was much stronger than Fe-PDA–MCM-41 and Zn-PDA–MCM-41.

**Figure 4.**
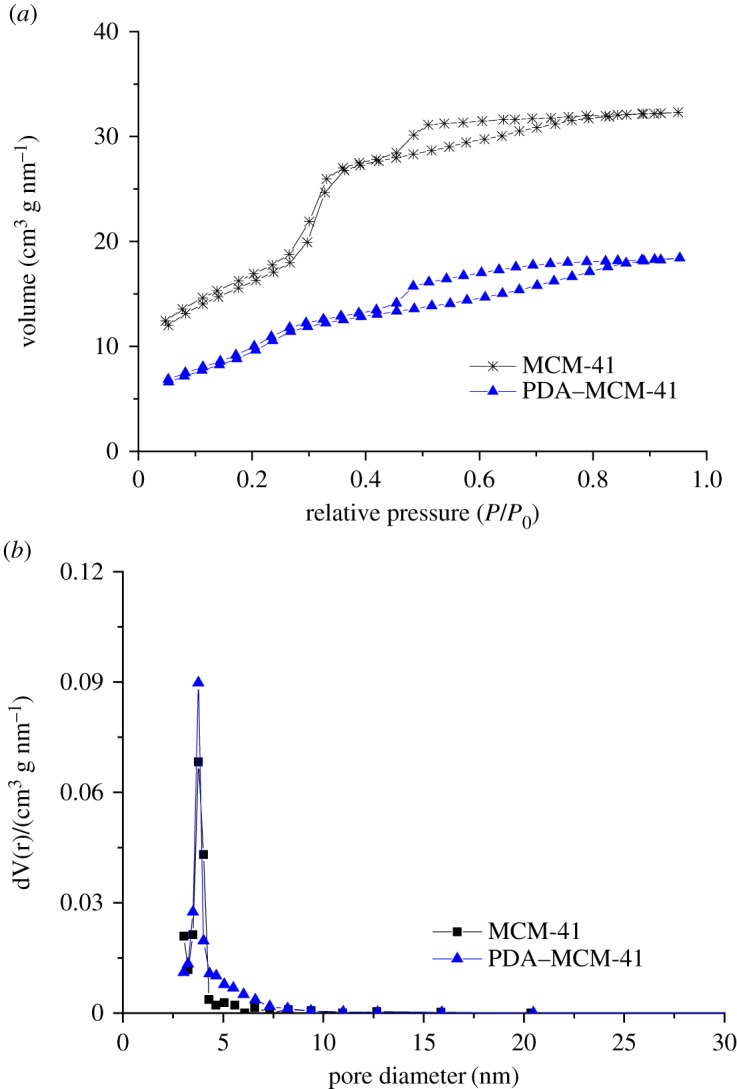
N_2_ adsorption/desorption isotherms (*a*) and pore size distribution (*b*) of MCM-41 and PDA–MCM-41.

As shown in figure 4*a*, the N_2_ adsorption/desorption isotherms of MCM-41 and PDA-Cu-MCM-41 belong to type IV (the slope of it was decreasing) with an H4 hysteresis loop (hysteresis loop was closed at *p*/*p*_o_ = 0.4), which confirmed their mesoporous structure according to the previous report [29]. The pore size distributed homogeneously from 3 to 4 nm for all the samples calculated by the Dollimore-Hill method as shown in figure 4*b*. What is more, PDA inclusion decreases the BET surface calculated by multi-point BET and significantly increases pore volume of mesoporous silica because the old pores were blocked and a new void area and new pores formed between PDA and mesoporous silica, but have slight effect on the pore size as shown in table 1.

**Table 1.** The pore structural parameter of MCM-41 and PDA–MCM-41.

sample	BET surface (m^2^ g^–1^)	pore volume (cm^3^ g^–1^)	pore size (nm)
MCM-41	986	0.243	3.76
PDA–MCM-41	828	0.318	3.76

**Figure 5.**
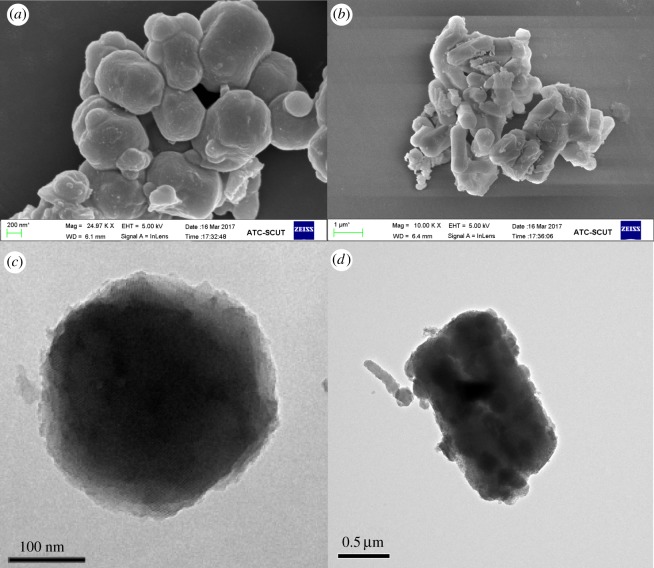
SEM images of MCM-41 (*a*), PDA–MCM-41 (*b*), and TEM images of MCM-41 (*c*), PDA–MCM-41 (*d*).

Figure 5 depicts the SEM and TEM images of MCM-41 and PDA–MCM-41. As shown, the regular hexagonal pore structure was well maintained without agglomeration for both samples, consistent with the XRD results. In figure 5*a*,*c*, the surface of MCM-41 was rough. After encapsulated by PDA, a thin layer was detected on the surface of samples as shown in figure 5*b*,*d*.

### 3.2. Adsorption performance

Table 2 lists the AC of various mesoporous silica. On the one hand, PDA encapsulation significantly increased the pore volume of mesoporous silica. On the other hand, the π–π interactions between the PDA layer and triazolone strengthened the interaction between triazolone and mesoporous silica. Therefore, the AC of the mesoporous silica increased significantly after encapsulated by PDA. The ferric and zinc ions further improved the AC of the samples owing to the bridge effect in which the metal ions coordinated with the PDA layer and triazolone, respectively, to strength the interaction between triazolone and mesoporous silica confirmed by the XPS analysis mentioned above. For example, the AC of Fe-PDA–MCM-41 increased up to 173 mg g^–1^, which was 160% more than that of MCM-41, while for Cu-PDA–MCM-41, the AC decreased owing to the excessively strong interaction between copper ion and triazolone as illustrated in the XPS analysis. On the other hand, the coordination between PDA and metal ions would be weakened resulting in the invalidation of the metal ion bridge effect for the PDA layer and triazolone.

**Table 2.** The AC of different types of mesoporous silica for triazolone.

sample	metal ion concentration (%)	AC (mg g^–1^)
MCM-41	0	108
PDA–MCM-41	0	120
Fe-PDA–MCM-41	0.41	173
Cu-PDA–MCM-41	1.19	95
Zn-PDA–MCM-41	2.44	141

### 3.3. Sustained release test

**Figure 6.**
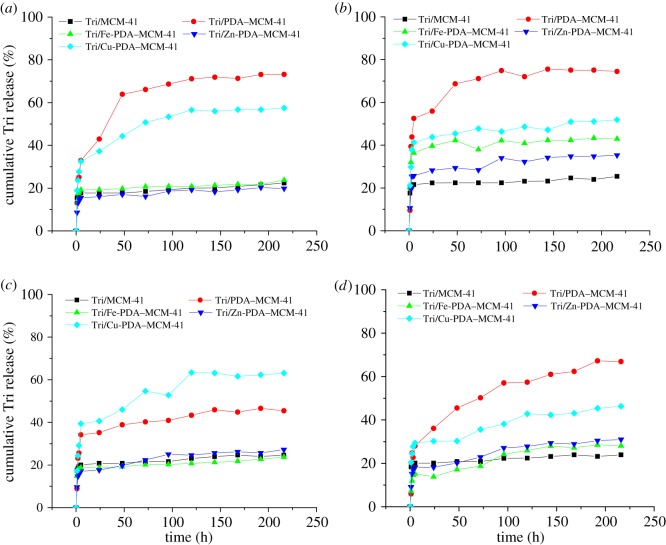
Effect of pH ((*a*) pH 7, (*b*) pH 1, (*c*) pH 4, (*d*) pH 10) on sustained release performance of the sustained released system.

Figure 6 depicts the sustained release curves of Tri/MCM-41, Tri/PDA–MCM-41, Tri/Zn-PDA–MCM-41, Tri/Cu-PDA–MCM-41 and Tri/Fe-PDA–MCM-41 at different pH values. Under natural conditions at pH 7, the release speed sequence judged by the variation of the Cumulative Tri release (%) in a certain period as shown in figure 6*a* was Tri/PDA–MCM-41 > Tri/Cu-PDA–MCM-41 > Tri/Fe-PDA–MCM-41 ≈ Tri/MCM-41 ≈ Tri/Zn-PDA–MCM-41; indicating that PDA encapsulation increased the release speed owing to the triazolone adsorbed by the PDA layer on the surface of the mesoporous silica which would be easily released into the solution. The PDA layer became compact after complexed with metal ions which increased the steric hindrance for the sustained release system. What is more, the coordination of metal ions strengthened the interaction between the PDA layer and triazolone through the coordination bond for zinc ion and ferric ion. As a result, the release speed decreased after metal ion coordination.

At different pH values, the release speed has no significant difference in the sustained release performance for Tri/MCM-41. After being encapsulated by PDA, the system became pH-sensitive. Under acid conditions as shown in figure 6*b*,*c*, the lower the pH was, the faster the release speed was for the samples encapsulated by PDA because the PDA membrane was unstable under acid conditions and tended to be hydrolysed. As a result, the triazolone adsorbed by the PDA layer would be released and the release speed was accelerated. Under basic conditions as shown in figure 6*d*, the release speed gets smaller owing to the protective effect of basic conditions on the PDA membrane to increase the steric hindrance. The results concluded that the system encapsulated by PDA was pH-sensitive with various sustained release performance at different pH values. For Tri/Cu-PDA–MCM-41 and Tri/Fe-PDA–MCM-41, the trend of changes at different pH values was similar to Tri/PDA–MCM-41, while for Tri/Zn-PDA–MCM-41, the interaction of zinc ion with hydroxyl ions was much stronger than with the PDA layer. As a result, the interaction with triazolone was weakened under basic conditions and the sustained released speed increased in contrast to Tri/PDA–MCM-41, Tri/Cu-PDA–MCM-41 and Tri/Fe-PDA–MCM-41.

### 3.4. Kinetics study

To further understand the sustained release mechanism, the data of triazolone sustained release from Tri/MCM-41, Tri/PDA–MCM-41 and Tri/Cu-PDA–MCM-41 at pH 7 were fitted to the zero-order model, first-order model, Higuchi model [34] and Korsmeryer–Pappas model, [35]. As shown in table 3, the drug release behaviour of sustained release particles was most consistent with the Korsmeryer–Pappas kinetic equation. The diffusion coefficient *n*th power for time (*t*) is 0.0543, 0.2880 and 0.0619 and calculated from the kinetic equation for Tri/MCM-41, Tri/PDA–MCM-41 and Tri/Fe-PDA–MCM-41, while all of them were below 0.45. The values obtained indicated that the sustained release of triazolone from the particles of metal- and PDA-loaded materials showed the same behaviour as pure MCM material which is controlled by a Fickian diffusion mechanism [36,37] and the difference of the concentration is the main impact on the release process. The results proved that PDA decoration or metal ion coordination only increases the steric hindrance and the interaction between carrier and triazolone instead of changing the original structure of the pure MCM material in accordance with XRD and BET analysis results mentioned above.

**Table 3.** Fitting results for drug release curves of Tri/MCM-41, Tri/PDA–MCM-41 and Tri/Cu-PDA–MCM-41 particles at pH 7.

kinetic model	Tri/MCM-41	Tri/PDA–MCM-41	Tri/Fe-PDA–MCM-41
zero-order			
*K*	0.0427	0.2794	0.0434
*R*^2^	0.3779	0.7061	0.3448
first-order			
*K*	−0.0015	−0.0081	−0.0016
*R*^2^	0.6446	0.5201	0.6330
Higuchi			
*K*	1.8512	6.1459	1.9619
*R*^2^	0.7910	0.7406	0.4711
Korsmeyer–Peppas			
*K*	15.483	17.613	15.960
*n*	0.0543	0.2880	0.0619
*R*^2^	0.8550	0.9487	0.8127

## 4. Conclusion

In this work, the sustained release systems of triazolone/PDA/MCM-41 mesoporous silica were prepared and its performance of AC and sustained release was further improved by coordination with metal ions. The characterization also confirmed the existence of coordination between triazolone and the PDA layer through the bridge effect of metal ions. The regular hexagonal pore structure and pore size were well maintained without agglomeration. The AC of Fe-PDA–MCM-41 increased up to 173 mg g^–1^, which was 160% more than MCM-41 without PDA decoration. What is more, the as-synthesized system encapsulated by PDA showed significant pH-sensitivity lying on the stability of PDA layer. Their sustained release curves at pH 7 could be described by the Korsmeyer–Peppas equation consistent with Fickian diffusion, indicating that the sustained release mechanism of mesoporous silica was not changed by PDA inclusion or metal ion coordination, and the original structure of the pure MCM material was well maintained. This sustained release system with pH-sensitivity can be well stored in the neutral or basic environment and activated in the acid environment which is expected to have tremendous application potentiality in the application of controllable pesticide delivery with different sustained release behaviour at different pH values.

## Supplementary Material

BET raw data

## Supplementary Material

FT-IR raw data

## Supplementary Material

XPS raw data

## Supplementary Material

XRD raw data

## Data accessibility

The raw data supporting this article have been uploaded as part of the electronic supplementary material.
